# Hypertrophic cardiomyopathy: insights into pathophysiology and novel therapeutic strategies from clinical studies

**DOI:** 10.1186/s43044-024-00600-4

**Published:** 2025-01-07

**Authors:** Samuel Oluwadare Olalekan, Olalekan Olanrewaju Bakare, Patrick Godwin Okwute, Ifabunmi Oduyemi Osonuga, Muinat Moronke Adeyanju, Victoria Biola Edema

**Affiliations:** 1 Department of Physiology, Faculty of Basic Medical Sciences, Obafemi Awolowo College of Health Sciences, Olabisi Onabanjo University, Sagamu Campus, Sagamu, Ogun State Nigeria; 2 Department of Biochemistry, Olabisi Onabanjo University, Sagamu Campus, Sagamu, Ogun State Nigeria; 3 Department of Physiology, Babcock University, Ilishan, Ogun State Nigeria

**Keywords:** Hypertrophic cardiomyopathy, Genetic therapy, Cardiac myosin inhibitors, Sarcomere proteins

## Abstract

**Background:**

Hypertrophic cardiomyopathy (HCM) is a frequently encountered cardiac condition worldwide, often inherited, and characterized by intricate phenotypic and genetic manifestations. The natural progression of HCM is diverse, largely due to mutations in the contractile and relaxation proteins of the heart. These mutations disrupt the normal structure and functioning of the heart muscle, particularly affecting genes that encode proteins involved in the contraction and relaxation of cardiac muscle.

**Main body:**

This review focused on understanding the role of contractile and relaxation proteins in the pathogenesis of hypertrophic cardiomyopathy. Mutations in contractile proteins such as myosin, actin, tropomyosin, and troponin are associated with hypercontractility and increased sensitivity of the heart muscle, leading to HCM. Additionally, impaired relaxation of the heart muscle, linked to abnormalities in proteins like phospholamban, sarcolipin, titin, myosin binding protein-C, and calsequestrin, contributes significantly to the disease. The review also explored the impact of targeted therapeutic approaches aimed at modulating these proteins to improve patient outcomes. Recent advances in therapeutic strategies, including novel pharmacological agents like mavacamten and aficamten, were examined for their potential to help patients manage the disease and lead more accommodating lifestyles.

**Conclusions:**

The review underscored the significance of early diagnosis and personalized treatment approaches in managing HCM. Future research should prioritize the development of robust biomarkers for early detection and risk stratification, particularly in diverse populations, to enhance clinical outcomes. Furthermore, it is imperative to delve deeper into the genetic mutations and molecular mechanisms associated with HCM, with a focus on exploring the roles of less-studied myocardial relaxation proteins and their interactions with sarcomere constituents.

## Background

Hypertrophic cardiomyopathy (HCM) is a diverse yet manageable heart condition with varying degrees of severity. It can lead to heart failure, atrial fibrillation (AF), and sudden arrhythmic death. This disease is marked by left ventricular (LV) hypertrophy that cannot be attributed to other causes and affects individuals of all ages and ethnicities. [1]. Dynamic left ventricular outflow tract (LVOT) obstruction is a hallmark and one of the determinants of the clinical course in HCM [2]. In China, the prevalence of HCM rose from 0.0069% in 2010 to 0.076% in 2019, with incidence increasing from 6.85 to 11.76 per 100,000 person-years [3]. Males showed higher prevalence than females, with a notable rise in heart failure cases among patients. Similarly, in Korea, prevalence increased from 0.016% in 2010 to 0.031% in 2016, with incidence climbing from 4.15 to 5.6 per 100,000 person-years, reflecting improved disease recognition [4]. The prevalence of HCM peaks in older adults, particularly in their 70 s, with common symptoms including chest pain, shortness of breath, and syncope. In the United States, HCM is estimated to affect 1 in 500 individuals, with obstructive HCM (oHCM) at 1.65 per 10,000 in 2016 [5]. Despite stable prevalence rates from 2016 to 2018, many patients remain undiagnosed, highlighting the need for better clinical identification and management strategies to enhance patient outcomes.

Wide variations exist in the disease on nearly all fronts, ranging from its genetic foundation to its clinical manifestation. In most cases, HCM is a monogenic disorder, making family screening advisable. [6]. More than 2000 mutations in genes encoding sarcomere proteins have been linked to HCM, which has an autosomal dominant inheritance pattern. The most commonly affected genes are encoding for contratile proteins such as beta myosin heavy chain and myosin-binding protein C [7]. Also, mutations in the genes encoding cardiac troponin T (TNNT2) and cardiac troponin I (TNNI3) have been linked to the development of HCM [8]. The mutations disrupt the normal structure and function of the sarcomere proteins, leading to abnormal myocardial hypertrophy [9]. HCM affects people of all ages and can have a significant impact on cardiac function and overall quality of life.

The pathophysiology of HCM involves the disruption of normal cardiac muscle structure and function due to sarcomere protein mutations. This leads to myocardial disarray, impaired relaxation, and abnormal contractile function. In its manifestation, HCM can be obstructive or non-obstructive. Obstructive HCM is characterized by the presence of a dynamic obstruction in the LVOT, caused by a thickened septum impeding blood ejection from the heart. This obstruction can lead to symptoms such as shortness of breath, and chest pain [10]. On the other hand, non-obstructive HCM involves hypertrophy of the heart muscle without significant blockage of blood flow. Patients with this type of HCM may experience symptoms like palpitations, fatigue, and difficulty with exercise tolerance [11].

While several studies have focused on the management of both types of HCM [11–13], it is pertinent to examine predictive factors of HCM, for early diagnosis and management. Also, several studies have considered the genetic and phenotypic manifestations of mutation in contractile proteins in the pathogenesis of HCM [7, 9]. This current study goes further to examine, the role of mutation in myocardial relaxation proteins such as phospholamban (PLN), sarcolipin (SLN), titin, Myosin Binding Protein-C (MyBP-C) and Calsequestrin in the pathology of HCM considering the genetic and phenotypic manifestations. By elucidating the complexities of HCM and highlighting potential novel therapies, this review serves as a resource for clinicians, guiding them in their approach to diagnosing and managing HCM in diverse patient populations. Overall, the findings from this review can inform future research directions and clinical practices, ultimately contributing to better health outcomes for individuals affected by HCM.

## Predictive factors in hypertrophic cardiomyopathy and associated disease conditions

Predicting the onset and progression of HCM is paramount for early detection and effective management of this complex cardiac disorder. Recent studies have endeavoured to identify predictive factors across various study groups and populations, shedding light on key determinants influencing HCM development and clinical outcomes. Recent studies have addressed factors predicting HCM in diverse study cohorts.

Xu et al. conducted a retrospective observational study targeting patients with HCM to identify clinical predictors of obstructive sleep apnea (OSA) [14]. They found that OSA was present in about 59% of the patients they studied. HCM patients with OSA were typically older, more often male, and had more clinical comorbidities, including hypertension, AF, and cardiac remodelling. Their research elucidated the feasibility of utilizing clinical characteristics to identify individuals at risk of OSA within the HCM population. By recognizing specific predictors associated with OSA, healthcare providers can implement early screening and intervention strategies to mitigate potential complications.

Kramer et al. undertook a prospective cohort study focusing on patients with HCM to elucidate predictors of major AF endpoints [15]. Through their investigation, they identified key factors contributing to the risk stratification for major AF events in individuals with HCM. These factors include older age, high BMI, moderate or severe mitral regurgitation (MR), history of arrhythmia, increased left atrial (LA) volume, and reduced LA contractile percentage. By understanding these predictors, clinicians can implement personalized monitoring and preventive measures to manage AF-related complications effectively.

Seo et al. conducted a retrospective analysis focusing on individuals with HCM to examine the presence of right ventricular (RV) involvement and its relationship with left ventricular structure and biventricular dysfunction. Their findings revealed that 14.4% of the patients exhibited RV involvement, which was associated with more severe cardiac conditions and a heightened risk of adverse events such as death or hospitalization. Furthermore, impaired RV strain, observed in patients with RV involvement, was associated with poorer outcomes, underscoring the significance of monitoring RV health in HCM patients for prognosis [16]. Their findings highlighted the prognostic significance of RV involvement in predicting adverse clinical events and impaired RV strain, thereby emphasizing the importance of comprehensive cardiac assessment in individuals with HCM.

In a retrospective study, She et al. explored the use of myocardial strain for detecting fibrosis in HCM patients through cardiac magnetic resonance. They discovered that segmental circumferential strain (SCS) and maximal wall thickness (MWT) were significant biomarkers for identifying myocardial fibrosis, with SCS showing the highest specificity when used alongside MWT [17].

Understanding the predictive factors for HCM across different study populations enhances the ability to diagnose and manage the condition early. Key factors such as OSA, AF endpoints, RV involvement, and myocardial fibrosis have been identified as significant in predicting adverse outcomes in HCM patients. Recognizing and monitoring these predictors allows for personalized and effective intervention strategies, ultimately improving patient outcomes and quality of life.

## The pathophysiology of hypertrophic cardiomyopathy considering the sarcomeric proteins

The heart’s ability to contract and relax efficiently is fundamental to its function in pumping blood throughout the body. This intricate process is orchestrated by a complex interplay of various proteins, including both contractile and relaxation proteins. In the context of cardiac health, understanding the roles of these proteins is paramount, particularly in conditions such as HCM, a genetic disorder characterized by abnormal thickening of the heart muscle.

### The role of contractile proteins in hypertrophic cardiomyopathy

Contractile proteins in the heart, such as myosin, actin, and titin, play a crucial role in the contractility and pumping function of the myocardium by facilitating interactions between myosin and actin regulated by calcium [18, 19]. Mutations in these proteins are linked to HCM, highlighting their significance in the disease’s pathogenesis and as potential therapeutic targets, as highlighted in Table 1 [20, 21].

**Table 1:** Cardiac contractile protein structure, function, and mutations in hypertrophic cardiomyopathy

Contractile protein	Structure	Subtypes/subunit	Function and role in the heart	Mutation	Implication of mutations	References
MYOSIN	It is a heterohexameric protein, that consists of Heavy chains (MHCs), Regulatory light chains (RLCs), and Essential light chains (ELCs)	α-isoform-predominantly expressed by the atrium β-cardiac myosin- expressed by the ventricles. It is encoded by the gene (MYH7)	Powers heart contraction, initiates cardiac contraction, shapes the mechanical environment	Isoform, switching of myosin light chain 2 (MLC2) Dysregulation of MLC2 isoforms MYH7 R403Q/ + variant is associated with β-cardiac myosin heavy chain (MYH7)- it causes hypercontractility of the heart	Mutation or abnormalities in myosin leads to cardiac diseases like HCM, due to the enlargement of cardiac muscle and hypercontractility	[20, 22–24]
ACTIN	Actin (thin) filaments are composed mainly of globular actin (G-actin) monomers that have aggregated to form long helical assemblies called filamentous actin ( F-actin) The *ACTC* gene encodes cardiac actin	α-cardiac actin (ACTC) β-actin (ACTB) γ-actin *(ACTG)*	Provides structural stability, initiates cardiac contraction by binding with myosin, influences cell migration, cell shape maintenance	A230V-ACTCmutation- it increases calcium sensitivity and leads to hypercontractile sarcomeres. cardiac α-actin p.A295S mutant- it is clinically shown to increase calcium sensitivity in the heart sarcomere E101K mutation of actin gene- it is particularly associated with apical HCM	Mutations or abnormalities of cardiac actin have been shown to clinically lead to hypersensitivity and hypercontractility of the heart sarcomere leading to HCM	[25–28]
TROPOMYOSIN	it is an α-helical coiled-coil protein that binds to actin	Consists of two isoforms in the myocardium alpha tropomyosin (αTpm) Kappa tropomyosin (κTpm)	Regulates cardiac muscle contraction, inhibits actin-myosin interaction, and controls access to actin-binding sites organization and dynamics of the actin cytoskeleton	Mutations occur at the C- and N-ends of Tpm M281T and I284V Tpm gene mutations are linked to HCM	These Mutations in tropomyosin lead to hypertrophic and dilated cardiomyopathies; alter Tpm’s affinity to F-actin; Affect systolic and diastolic function of the heart	[19, 29–33]
TROPONIN	A thin filament protein made up of three subtype molecules	Cardiac Troponin I (TnI) (TNNI3 gene encodes troponin I) Cardiac Troponin T (TnT) (TNNT2 gene encodes Troponin T Troponin C (TnC)	Regulates muscle contraction and relaxation by responding to changes in calcium ion concentration; TnC binds to calcium inducing conformational changes in the troponin complex TnI inhibits the binding of actin to myosin in the absence of calcium TnT binds to tropomyosin and anchors troponin complex to actin thin filament	TNNI3R21C/ + Mutation- increases calcium binding of cardiac troponin and the affinity of cardiac troponin C for cardiac troponin I in solution TNNT2R92Q/ + variant- increases calcium sensitivity, this high sensitivity changes tropomyosin positioning, and contributes to diastolic dysfunction	Mutation in cardiac troponin genes (TnI and TnT) accounts for a high percentage of HCM The mutations also lead to diastolic dysfunction	[8, 34–36]

The contractile proteins in the heart, play crucial roles in cardiac muscle function. Myosin, a heterohexameric protein, consists of Myosin Heavy Chains (MHCs), Regulatory light chains (RLCs), and Essential light chains (ELCs). Mutations in the MYH7 gene, such as the MYH7 R403Q/ + variant, lead to hypercontractility, contributing to HCM. Actin, composed of ACTC gene-encoded α-cardiac actin (ACTC) and others, influences cardiac contraction and shape. Mutations, like A230V-ACTC, clinically increase calcium sensitivity, resulting in hypercontractile sarcomeres. Tropomyosin regulates muscle contraction, and mutations like M281T and I284V, are associated with HCM. Troponin, with subtypes TnI, TnT, and TnC, responds to calcium changes. Mutations, such as TNNI3R21C/ + , increase calcium binding, contributing to HCM, and TNNT2R92Q/ + variant heightens calcium sensitivity, affecting tropomyosin positioning and causing diastolic dysfunction. Mutations in troponin genes often lead to HCM and diastolic dysfunction. These structural alterations highlight the crucial link between contractile protein mutations and various cardiac diseases.

### The role of relaxation proteins in hypertrophic cardiomyopathy

Cardiac muscle relaxation plays a crucial role in the cardiac cycle, and its significance extends beyond being a passive aftermath of contraction. It is indispensable for optimal ventricular filling between heartbeats. While the heart’s contraction may be robust, an excessively slow relaxation phase can impede proper ventricular filling, leading to incomplete filling and reduced diastolic volume at the beginning of the next contraction [37].

Even with normal contractile function, slow relaxation adversely affects cardiac output [38]. To enhance treatments for cardiac diseases associated with impaired muscle relaxation, a more profound comprehension of the regulatory mechanisms governing cardiac muscle relaxation is essential.

Understanding relaxation proteins is crucial for comprehending cardiac function and their role in the pathogenesis of cardiac dysfunctions and diseases such as HCM as expatiated in Table 2. Phospholambam and SLN regulate calcium uptake by sarcoplasmic reticulum ATPase. Titin modulates muscle contractility and myosin-binding protein-C influences cardiac contractility. Calsequestrin serves as a key calcium regulator in the sarcoplasmic reticulum.

**Table 2:** Cardiac relaxation proteins structure, function, and mutations in hypertrophic cardiomyopathy

Relaxation proteins	Structure/composition	Functions	Regulations	Implications of mutation	References
Phospholamban (PLN)	A 52 amino acid protein found in the sarcoplasmic reticulum	Regulates SERCA, and inhibits Ca2 + uptake during diastole. PLN acts by binding to the transmembrane domain and decreases the SERCA pump’s affinity for Ca2 +	Regulated by phosphorylation, cAMP-dependent PKA and CaMKII	Known human mutations include R14del, R9C, R9L, R9H, Leu-39stop, and R25. The Mutations disrupt calcium homeostasis and also induce cardiomyopathy	[39–42]
Sarcolipin (SLN)	31-residue tail-anchored integral membrane protein, also found is the SR membrane	Inhibits SERCA, decreases calcium uptake, decreases, contractility induces relaxation	Regulated by adrenergic signaling and reversible phosphorylation	SLN overexpression abnormality alters PLB expression, causing Ca2 + handling alteration	[43–45]
Titin	Giant filament protein, anchored in Z-disc and M-lines. Consist of two isoforms which are N2BA (3.2–3.3 MDa) and N2B (3.0 MDa)	Acts as a molecular spring that regulates muscle contraction	Dynamically modulated by posttranslational modifications	Mutations in the TTN gene are linked to various muscular and cardiac disorders, such as dilated cardiomyopathy (DCM) and different myopathies. These mutations can disrupt the protein’s structural integrity and affect the passive tension it provides to cardiac myocytes, leading to increased myocardial stiffness and impaired relaxation of the heart during diastole	[46–50]
Myosin Binding Protein-C (MyBP-C)	It is a flexible, rod-like protein with a molecular weight of approximately 140 kD. It is encoded by three orthologous genes specific to different muscle fibre types: fast-type, slow-type, and cardiac. The cardiac isoform, known as cardiac MyBP-C (cMyBP-C), consists of 11 domains, including Ig-like and Fn3-like domains, labelled C0 through C10. This protein is situated within the C-zone of the sarcomere. The C-terminal domains (C8–C10) anchor cMyBP-C to the thick filament backbone, while the N-terminal domains (C0–C2) play a crucial role in modulating cardiac contractility	It modulates cardiac contractility by interacting with the myosin head region and/or actin MyBP-C works as either a brake for the sliding filament or an activator of the tropomyosin-troponin, reducing Ca2 + sensitivity of force and rates of force development	It is regulated through phosphorylation	MyBP-C dephosphorylation which occurs in heart failure and genetic cardiomyopathies is linked to diastolic dysfunction	[51–53]
Calsequestrin	A key calcium ion binding protein located in the sarcoplasmic reticulum, it attaches to the junctional SR membrane through interactions with membrane proteins and can reversibly polymerize as calcium concentration rises	A crucial element of excitation–contraction coupling, it acts as a Ca2 + reservoir, buffer, and regulator of Ca2 + release channels	Oligomerization	Mutations associated with Catecholaminergic Polymorphic Ventricular Tachycardia (CPVT) and malignant hyperthermia/environmental heat stroke (MH/EHS) arrhythmias include those in genes encoding proteins such as ryanodine receptor type 2 (RYR2) and calsequestrin (CASQ2)	[54, 55]

Mutations and abnormalities in these proteins are associated with diverse disorders including HCM diastolic cardiomyopathy (DCM)and arrhythmias.

### The role of phenotype-based genetic testing for hypertrophic cardiomyopathy in the current practice

Phenotype-based genetic testing plays a significant role in diagnosing and managing hypertrophic cardiomyopathy (HCM) [56]. This approach uses clinical and echocardiographic markers to predict the likelihood of a positive genetic test result, which can guide patient management and family screening. Over the past decades, advances in genetic testing and phenotyping have transformed the diagnostic, prognostic, and therapeutic approaches to HCM [56]. A phenotype-based genetic testing strategy remains integral to clinical practice, enabling tailored management plans for affected individuals and at-risk relatives.

HCM is predominantly inherited in an autosomal dominant pattern, with mutations in genes encoding sarcomeric proteins, such as *MYH7* (beta-myosin heavy chain), *MYBPC3* (myosin-binding protein C), *TNNI3* (troponin I), and *TNNT2* (troponin T) accounting for the majority of cases. While genetic testing identifies the causal mutations in 40–60% of HCM patients, phenotyping remains the cornerstone for initial diagnosis. This involves integrating clinical evaluation, imaging (primarily echocardiography and cardiac MRI), and functional assessments to characterize the disease’s structural and functional abnormalities [57].

Phenotyping in HCM allows clinicians to distinguish sarcomeric HCM from phenocopies like amyloidosis, Fabry disease, or mitochondrial myopathies. This differentiation is crucial, as non-sarcomeric forms may respond to alternative therapies and have distinct implications for prognosis [58]. Emerging evidence suggests that genetic mutations influence disease progression and therapeutic response. For instance, patients with *MYBPC3* mutations often exhibit late-onset HCM, while those with *MYH7* mutations may show more severe hypertrophy and earlier presentation. Such insights are increasingly being used to guide therapeutic strategies, including pharmacological agents [59, 60].

## Therapeutic approaches in the treatment of hypertrophic cardiomyopathy

The management of HCM involves a multifaceted approach, utilizing medications that target different aspects of the disease to alleviate symptoms and prevent complications. This includes the use of beta-blockers, calcium channel blockers, and antiarrhythmic drugs, each with distinct mechanisms of action and therapeutic effects. In some cases, invasive procedures like septal myectomy or alcohol septal ablation may be necessary to relieve LVOT obstruction. ICDs are utilized to prevent sudden cardiac death in patients at high risk [61].

Beta-blockers, such as metoprolol and atenolol, play a significant role in managing hypertrophic cardiomyopathy (HCM) by reducing heart rate and myocardial oxygen consumption, thus alleviating symptoms like chest pain and shortness of breath. Metoprolol, a β-adrenergic receptor blocker, has been shown to effectively reduce LVOT gradients, leading to symptomatic relief and improved quality of life in patients with obstructive HCM [62]. However, it does not significantly enhance exercise capacity or peak oxygen consumption, indicating that while metoprolol is beneficial for symptom management, its effects on overall physical performance are limited. Atenolol, another β-adrenergic blocker, has been studied in both human and veterinary medicine. Research on cats with subclinical HCM revealed that atenolol reduces heart rate and rate-pressure product but does not significantly improve quality of life or activity levels, and its use can be associated with severe bradyarrhythmias, underscoring the need for careful monitoring during treatment [63, 64].

Calcium channel blockers (CCBs) such as verapamil and diltiazem are also used in HCM management due to their ability to inhibit calcium influx into myocardial cells, thereby improving diastolic function and reducing LVOT obstruction [65]. Verapamil has been shown to alleviate symptoms such as dyspnea and chest pain, and to improve exercise capacity by reducing myocardial contractility and LVOT gradients. However, its effects can be variable, and it carries the risk of hypotension, which may exacerbate symptoms in some patients [65, 66]. Diltiazem, another CCB, also reduces myocardial contractility and heart rate, but its effectiveness in reversing the HCM phenotype is limited. Moreover, it carries risks such as severe hypotension and bradycardia, emphasizing the importance of careful patient selection and monitoring [67].

In addition to CCBs, calcium desensitizers are emerging as a promising class of pharmacologic agents designed to normalize calcium sensitivity, improving myocardial relaxation and reducing the hypercontractile state. These agents work by modulating the interaction of calcium with troponin, restoring balance in sarcomeric function. By targeting sarcomeric hypercontractility, a primary driver of HCM progression, calcium desensitizers reduce excessive contractile force, thereby improving diastolic function without impairing systolic function [68]. Moreover, reducing hypercontractility decreases the metabolic demand on cardiomyocytes, mitigating cellular stress, energy depletion, and long-term myocardial remodeling. Nebivolol, a beta-adrenoceptor antagonist, has also demonstrated calcium desensitizing effects, though these were more pronounced in mouse models than in human cardiac strips [69]. Early-stage calcium desensitizers have shown promise in preclinical models of HCM, improving myocardial relaxation and reducing fibrosis.

Antiarrhythmic drugs, including amiodarone and disopyramide, are crucial in managing arrhythmias commonly associated with HCM, which can lead to sudden cardiac death. Amiodarone, effective in maintaining sinus rhythm in atrial fibrillation (AF) patients, has been shown to significantly reduce postoperative AF incidence after septal myectomy in patients with obstructive HCM [70]. However, its use is associated with significant adverse effects, including QT interval prolongation, thyroid dysfunction, and hepatic toxicity, necessitating careful monitoring during long-term therapy [71, 72]. Disopyramide, a class Ia antiarrhythmic, is particularly used in managing LVOT obstruction by reducing myocardial hypercontractility, thereby alleviating symptoms. Despite its effectiveness, disopyramide is underutilized due to its side effects, such as anticholinergic effects and pro-arrhythmic risks, which require careful consideration in clinical practice [73, 74].

Effective management of HCM therefore has gone beyond the use of pharmacologic interventions like beta-blockers and calcium channel blockers but also emerging therapies such as myosin ATPase inhibitors and potentially, genetic therapies. Advances in diagnostic techniques and the development of novel therapeutic approaches hold promise for improving the prognosis and quality of life for individuals with HCM [75, 76]. Moreover, incorporating lifestyle modifications aimed at improving cardiometabolic health is essential for optimizing the prognosis and quality of life for individuals with HCM [77–80].

### Genetic therapy

Genetic therapy offers promising avenues for addressing HCM, a prevalent genetic disorder primarily caused by mutations in sarcomere protein genes like MYBPC3 and MYH7 [81, 82]. These mutations lead to unexplained left ventricular hypertrophy, diastolic dysfunction, and myocardial disarray. Recent studies highlight the potential of gene therapy in treating HCM. For instance, studies utilizing adenine base editors (ABE8e) and Cas9 nucleases delivered by AAV9 vectors have shown promise in correcting or inactivating pathogenic variants associated with HCM, preventing disease progression in preclinical models [83]. While ABE8e-based therapy demonstrated efficient correction in ventricular cardiomyocytes with durable effects, Cas9 nuclease therapy effectively silenced pathogenic alleles, albeit with dose-dependent toxicities, emphasizing the need for careful dosage regulation in clinical translation [83].

However, while genetic therapy presents a promising approach, challenges such as dose-dependent toxicities and immunologic reactions have been observed in preclinical trials [84]. Moreover, genetic testing remains complex, with interpretation of results and variant classification posing challenges in clinical implementation [85]. Nevertheless, the burgeoning field of genetic therapy holds great potential for revolutionizing the management of HCM by targeting its underlying genetic causes and offering personalized treatments tailored to individual genotypes and phenotypes [84, 85]. Further research and clinical trials are warranted to address safety concerns and optimize the translation of genetic therapy into clinical practice for the benefit of HCM patients.

In recent studies on HCM, significant strides have been made in understanding its nature and potential therapeutic interventions. HCM is characterized by the hypertrophy and disorganization of myocytes, often accompanied by interstitial fibrosis. Genetic screening and familial assessment are pivotal in managing HCM, along with strategies aimed at symptom control and preventing sudden cardiac death. Traditionally, septal reduction therapy and heart transplantation were the mainstays of disease management, but emerging pharmacotherapies are showing promise in clinical trials. For instance, gene therapy targeting specific genetic mutations associated with HCM, such as decreased expression of cardiac myosin-binding protein C (cMyBPC), has shown therapeutic potential. A study utilizing AAV9 gene transfer of cMyBPC N-terminal domains demonstrated significant improvement in cardiac function and reduced histopathological signs of cardiomyopathy, suggesting a promising mechanism for addressing the underlying pathology of HCM [86].

Moreover, research into long non-coding RNAs (lncRNAs) has shed light on their role in the pathophysiology of cardiac hypertrophy, offering potential therapeutic targets. Abnormal expression of lncRNAs has been linked to impairments in sarcomere function, intracellular calcium handling, and mitochondrial metabolism, all contributing to cardiac hypertrophy. Targeting these lncRNAs through gene therapy approaches holds promise for preventing or reversing cardiac hypertrophy and improving patient outcomes [87]. However, despite these advancements, challenges remain, such as the difficulty in diagnosing certain genetic forms of HCM, such as Anderson-Fabry disease (AFD), which can masquerade as idiopathic HCM. AFD is a lysosomal storage disorder associated with a specific form of HCM, making timely diagnosis crucial for implementing disease-modifying therapies. The role of cardiologists in the multidisciplinary management of AFD, from differential diagnosis to treatment initiation, is pivotal in optimizing patient care and outcomes [88].

These studies underscore the evolving landscape of HCM management, with genetic therapies, targeted pharmacotherapies, and multidisciplinary approaches offering hope for improved outcomes and quality of life for patients with this complex cardiovascular disorder.

### Novel pharmacological agents

#### Mavacamtem

Mavacamten (MYK-461) is a novel cardiac-specific small molecule developed by MyoKardia, designed to target the sarcomere by modulating the β-MHC. It works by reversibly inhibiting the binding between β-MHC and actin, thereby addressing the underlying pathophysiology of HCM. This mechanism reduces actin-myosin cross-bridge formation, which effectively mitigates hypercontractility and left ventricular stiffness. Studies highlighting the effectiveness of MYK-461 are addressed in Table 3.

**Table 3:** Overview of Mavacamten treatment interventions in clinical studies for hypertrophic cardiomyopathy

Study	Nature of HCM	Mechanism of Action of Treatment	Positive Outcome	Negative Outcome/Limitation	References
Phase 3, Randomized, Double-blind, Placebo-controlled Trial	Symptomatic obstructive HCM (HOCM)	Cardiac myosin inhibition	Enhanced exercise capacity, reduction in LVOT obstruction, improvement in New York Heart Association (NYHA) functional class, and better health status were observed	Treatment-related adverse events were mostly mild; only one patient in the placebo group experienced sudden death	[89]
Secondary Analysis of the EXPLORER-HCM Randomized Trial	Symptomatic HOCM	Cardiac myosin inhibition	Improved cardiopulmonary exercise testing parameters beyond peak oxygen consumption	N/A	[13]
Phase 3, Randomized, Double-blind, Placebo-controlled Trial	Symptomatic HOCM	Cardiac myosin inhibition	Markedly improved health status compared with placebo	Health status gains returned to baseline after treatment was stopped	[12]
Phase 2, Multicenter, Double-blind, Placebo-controlled Trial	Symptomatic nonobstructive HCM	Reversible inhibitor of cardiac-specific myosin	Generally well-tolerated, with a notable decrease in N-terminal pro-B-type natriuretic peptide (NT-proBNP) and cardiac troponin I (cTnI)	However, some participants experienced serious adverse events, including a reversible reduction in left ventricular ejection fraction (LVEF)	[11]
Randomized, Double-blind, Placebo-controlled Trial	Symptomatic HOCM referred for septal reduction therapy	Cardiac-specific myosin inhibitor	Resulted in a decrease in the proportion of patients meeting guideline criteria for septal reduction therapy	However, the long-term effects and outcomes are yet to be determined	[90]
Phase 3 Randomized Controlled Trial	Symptomatic patients with obstructive HCM	Cardiac myosin inhibitor	Enhancement in left ventricular diastolic function and resolution of systolic anterior motion were noted. Additionally, there was a decrease in LVOT obstruction, left atrial volume index, and the ratio of early mitral inflow velocity to lateral mitral annular early diastolic velocity	None reported	[91]
Post-hoc analysis of a clinical trial	Patients with obstructive HCM with or without hypertension	Cardiac myosin inhibitor	Clinical benefits of mavacamten were similar in patients with and without hypertension	None reported	[92]
Interim results of a long-term extension study	Symptomatic HOCM	Cardiac myosin inhibitor	Significant and long-lasting enhancements were observed in LVOT gradients, NT-proBNP levels, and NYHA functional class, indicating clinically relevant improvements	Adverse events included cardiac failure and atrial fibrillation, transient reductions in LVEF	[93]
Observational Study	Hypertrophic obstructive cardiomyopathy	Comparison of real-world HOCM patients with those enrolled in EXPLORER-HCM	About half of real-world HOCM patients were eligible for mavacamten treatment	Lack of direct comparison with placebo or other treatments	[94]
Longitudinal Observational Study	Symptomatic HOCM	Improvement in LVOT gradients, exercise capacity, symptoms	Sustained improvements in outcomes over > 3 years	Mild/moderate treatment-emergent adverse events	[95]
Phase 3, Double-blind, Placebo-controlled Trial	Patients with HOCM	Reduction in LVOTO, improvement in functional parameters and health status	Significant improvements in primary and secondary endpoints	Safety profile similar to placebo	[96]
Phase 2 Trial	HCM without outflow obstruction	Selective inhibitor of cardiac myosin ATPase	Reduction in NT-proBNP and troponin I	Significant reduction in outflow gradient, improved exercise capacity, quality of life	[97]

#### Aficamtem

Aficamten, a cardiac myosin inhibitor, selectively targets cardiac myosin, reducing actin-myosin cross-bridges responsible for hypercontractility in HCM [98]. In a prospective, randomized, controlled crossover study, researchers explored the effects of targeting actin-myosin interactions using aficamten to alleviate the overactivity of these protein interactions in HCM. The study showed dose-dependent reductions in left ventricular (LV) systolic function and LVOT pressure gradient. However, it was limited by a small sample size and was conducted on a feline model, which may not fully translate to human patients [99].

The REDWOOD-HCM Phase II trial evaluated aficamten;’s safety, tolerability, and dose-dependent reduction in myocardial contractility in patients with symptomatic HOCM. Aficamten’s targeted action on cardiac myosin selectively reduces actin-myosin cross-bridges, effectively addressing hypercontractility. Its shallow exposure–response relationship allows for personalized dosing, minimizing the risk of left ventricular ejection fraction (LVEF) reduction. The study found that aficamten had a favourable safety profile, with no treatment-related serious adverse events and no patients discontinuing due to adverse events. Adverse events were comparable to those seen with placebo, underscoring aficamten’s safety. No serious adverse events led to early termination, and any reduction in LVEF below 50% was reversible, suggesting aficamten’s promise as a therapeutic option for symptomatic HOCM [100]. Despite these overall positive outcomes, some patients experienced persistent symptoms, reflecting the complex and dynamic nature of HCM. The study faced several limitations, including short treatment duration, lack of functional capacity measures, and genetic assessment. The FOREST-HCM extension aims to address these issues with a five-year plan. While the initial study had its constraints, aficamten showed potential in lowering LVOT gradients and improving symptoms, warranting further investigation in Phase 3 trials as a potential therapeutic intervention for hypertrophic cardiomyopathy.

## Conclusions

This review focused mainly on understanding the role of contractile and relaxation proteins in the pathogenesis of hypertrophic cardiomyopathy. It also elucidated the major impact of the development of targeted therapeutic approaches aimed at modulating the target proteins in the improvement outcomes in patients with HCM. Mutations in contractile proteins such as myosin, actin, tropomyosin, and troponin have been associated with the development of hypercontractility and high sensitivity of the heart muscle, leading to hypertrophic cardiomyopathy. Additionally, impaired relaxation of the heart muscle, attributed to abnormalities in relaxation proteins like phospholamban, sarcolipin, titin, myosin binding protein-C, and calsequestrin, contributes to the pathogenesis of HCM.

Future research should prioritize the development and validation of robust biomarkers for early detection and risk stratification of HCM patients, particularly in diverse populations, to significantly improve clinical outcomes. Additionally, delving deeper into genetic mutations and molecular mechanisms associated with HCM remains imperative, with a particular emphasis on investigating the roles of less-explored myocardial relaxation proteins and their interactions with sarcomere constituents.

## Data Availability

All data in this study are included in this study.

## Abbreviations

HCM: Hypertrophic cardiomyopathy
LVOT: Left ventricular outflow tract
TNNT2: Cardiac troponin T
TNNI3: Cardiac troponin I
PLN: Phospholamban
SLN: Sarcolipin
MyBP-C: Myosin binding protein-C
AF: Atrial fibrillation
MHCs: Myosin heavy chains
RLCs: Regulatory light chains
ELCs: Essential light chains
ACTC: α-Cardiac actin
ACTB: Beta actin
ACTG: Gamma actin
Tpm: Tropomyosin
TnI: Troponin I
TnT: Troponin T
TnC: Troponin C
SERCA: Sarcoplasmic reticulum Ca^2^⁺-ATPase
cAMP: Cyclic adenosine monophosphate
PKA: Protein kinase A
CaMKII: Ca^2^⁺/Calmodulin-dependent protein kinase II
TTN: Titin
DCM: Dilated cardiomyopathy
CPVT: Catecholaminergic polymorphic ventricular tachycardia
RYR2: Ryanodine receptor type 2
CASQ2: Calsequestrin 2
ICD: Implantable cardioverter-defibrillator
QOL: Quality of life
CCB: Calcium channel blocker
LVOTO: Left ventricular outflow tract obstruction
TA-BSM: Transapical beating-heart septal myectomy
HOCM: Hypertrophic obstructive cardiomyopathy
r-LVR: Left ventricular reverse remodeling
HGAVB: High-grade atrioventricular block
CIED: Cardiovascular implantable electronic device
MYBPC3: Myosin binding protein C3
MYH7: Myosin heavy chain 7
ABE8e: Adenine base editor
AAV9: Adeno-associated virus serotype 9
cMyBPC: Cardiac myosin binding protein C
lncRNAs: Long non-coding RNAs
AFD: Anderson-fabry disease
NYHA: New York heart association
